# Environmental greenness, physical activity, and their synergistic effects on vital capacity weight index in children and adolescents exposed to PM_2.5_ and O_3_ in economically developed provinces of China

**DOI:** 10.1186/s12889-025-24439-9

**Published:** 2025-10-09

**Authors:** Zhiying Song, Xinli Song, Li Chen, Jianuo Jiang, Yi Zhang, Jieyu Liu, Ruolin Wang, Yang Qin, Ziqi Dong, Tongjun Guo, Wen Yuan, Peijin Hu, Tianjiao Chen, Guangrong Zhu, Jun Ma, Yanhui Dong, Yi Song

**Affiliations:** Institute of Child and Adolescent Health, School of Public Health, Peking University, National Health Commission Key Laboratory of Reproductive Health, No. 38 Xueyuan Rd, Haidian District, 100191 Beijing, China

**Keywords:** Vital capacity weight index, Air pollution, Greenness, Physical activity, Children and adolescents

## Abstract

**Background:**

The Vital Capacity Weight Index (VCWI) serves as a pivotal indicator of cardiopulmonary function among children and adolescents, reflecting their tissue oxygenation capacity and athletic potential. This study delves into the influence of environmental greenness and physical activity on VCWI in children residing in China’s economically prosperous provinces, who are exposed to air pollutants, namely PM_2.5_ and O_3_.

**Methods:**

We performed a cross-sectional analysis using data from the 2019 Chinese National Survey on Students’ Constitution and Health (CNSSCH), involving 62,987 students from the top eight provinces by GDP. Exposure to PM_2.5_ and O_3_ was estimated using data from the Tracking Air Pollution in China (TAP) platform. Greenness surrounding schools was assessed based on the China Land Cover Dataset (CLCD). Daily physical activity duration was used to classify participants into moderate or vigorous activity groups. Associations with VCWI were examined using univariate and multivariate logistic regression models. Interaction effects between air pollution and greenness or physical activity were assessed using additive models.

**Results:**

Higher concentrations of PM_2.5_ and O_3_ were significantly associated with lower VCWI. In contrast, greater greenness coverage and engagement in vigorous physical activity were linked to better VCWI outcomes. Interaction analysis showed that increased greenness may enhance the protective effect of lower air pollution levels on VCWI, while the interaction between physical activity and air pollution was not statistically significant.

**Conclusions:**

These findings highlight the potential of green environments and active lifestyles in buffering the negative respiratory effects of air pollution among children. The results provide evidence to inform integrated urban planning and public health initiatives aimed at improving children’s lung health.

**Supplementary Information:**

The online version contains supplementary material available at 10.1186/s12889-025-24439-9.

## Introduction

Forced vital capacity (FVC) is a key metric for assessing respiratory function and has long been used to monitor the physical health of primary and secondary school students in China [1–3]. The Vital Capacity Weight Index (VCWI), calculated as FVC (mL) divided by body weight (kg), offers a size-adjusted indicator of lung function, providing insights into both oxygen delivery capacity and physical fitness [4–6]. Due to its clinical relevance, VCWI is routinely included in national fitness surveillance reports for Chinese youth and is widely used to assess their physical development [7].

With rapid urbanization and industrialization, air pollution has become a major environmental health concern in China [8]. Fine particulate matter (PM_2.5_) and ground-level ozone (O_3_) are the two predominant air pollutants, both of which have been linked to adverse respiratory, cardiovascular, and metabolic health outcomes in previous studies [9–15]. School-age children are especially vulnerable, as exposure to these pollutants has been associated with impaired lung development and increased respiratory symptoms [8, 16–18]. Similar associations have been reported in studies from other Asian countries [19–22]. Beyond respiratory health, exposure to air pollution has also been linked to impaired cognitive development in children [23], and increased psychological stress and negative emotions among teachers [24], further highlighting its broader health impacts in school environments. Environmental greening factors, such as forests and grasslands, can effectively mitigate the concentrations of PM_2.5_ and O_3_ through the absorption and sedimentation effects of plants, thereby enhancing air quality [25–30]. Additionally, regular physical activity plays a crucial role in improving cardiopulmonary function, increasing vital capacity, and promoting the growth, development, and physical health of children and adolescents [29, 31].

Despite the importance of VCWI in assessing the physical health of children and adolescents, both domestic and international research on this topic remains limited [30]. In particular, studies on the improvement of VCWI under air pollution exposure through greenness and physical activity are scarce. To address this gap, the present study aims to examine the association between air pollutant exposure and VCWI, and to explore whether environmental greenness and physical activity modify this relationship. Using large-scale data from economically developed regions in China, the findings are expected to inform public health and environmental policy strategies that support the healthy growth of children.

## Methods

### Study design and participants

This study is based on a cross-sectional analysis using data from the 2019 cycle of the Chinese National Survey on Students’ Constitution and Health (CNSSCH), a long-standing nationwide surveillance system established in 1985. This collaborative initiative involves multiple government departments, including the Ministry of Education, the Ministry of Health, the Ministry of Science and Technology, the State Ethnic Affairs Commission, and the State Sports General Administration of the People’s Republic of China. The survey adopts a multi-stage stratified cluster sampling design, encompassing 31 provinces in mainland China, with the exception of Hong Kong, Macau, and Taiwan. Previous publications have detailed the survey’s methodologies and findings [32–34].

**Fig. 1:**
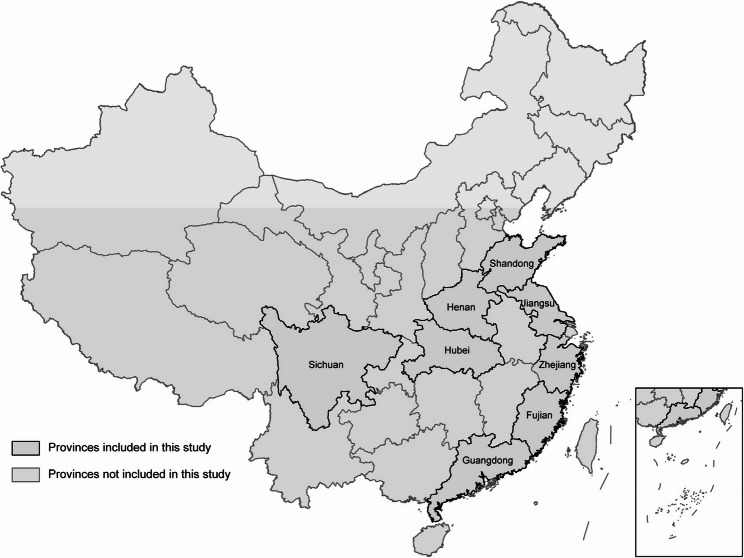
Geographical distribution of the eight provinces included in this study

To reduce regional socioeconomic confounding, we focused on data from the eight provinces with the highest gross domestic product (GDP) in 2019 (each exceeding 4 trillion yuan, Fig. 1) [35]. Participant recruitment was executed using a stratified cluster sampling method, which involved the random selection of classes across various grades within chosen schools. This approach aimed to capture a representative sample while mitigating the potential confounding effects of regional economic heterogeneity.

### Data processing and sample size determination

In the 2019 wave of the CNSSCH, data were collected from 273,169 school-aged participants. To ensure analytical accuracy, we applied data cleaning procedures that excluded individuals older than 18 years, as well as those with extreme values (Z-scores > 5), which may indicate measurement or reporting errors. After these exclusions, 227,601 valid observations remained. For the purposes of this study, we further restricted the sample to the eight provinces with the highest gross domestic product (GDP) in 2019, yielding a final analytic sample of 62,987 children and adolescents. This targeted dataset allows for focused analysis of health patterns among youth in China’s most economically developed areas.

### Ethical approval and Participant Protection

Ethical approval was obtained from the Institutional Review Board of Peking University Health Science Center (IRB00001052–19095). This approval underscores the commitment to safeguarding the rights and well-being of all research participants. During the data collection phase in schools, the principal plays a pivotal role in executing formal agreements to guarantee informed consent. This process encompasses diverse methods, including written and verbal consent, active consent, and passive consent (i.e., participants and their guardians were informed in advance, and absence of objection was considered as consent), obtained from both the children and their parents or guardians. Prior to initiating data analysis, stringent measures were taken to anonymize and de-identify all participant-related information, ensuring the confidentiality and privacy of the study population.

### Assessment of PM_2.5_ and O_3_ exposure

Ambient air pollutant concentrations for PM_2.5_ and O_3_ were derived from the “Tracking Air Pollution in China” (TAP) database (http://tapdata.org.cn/), which integrates satellite remote sensing, ground monitoring, emissions inventories, and chemical transport models [36]. In the present research, PM_2.5_ data were analyzed at a spatial resolution of 1 km [37], while O_3_ data were examined at a resolution of 10 km [38]. Annual pollutant exposure was estimated for each student based on the geographic coordinates of their school. We used the average concentration over the three years preceding the survey (2017–2019) to approximate long-term exposure. Since the CNSSCH did not include participants’ home addresses, school location was used as the exposure reference point. This approach is widely adopted in population-based studies in China, where students spend most of their daytime at school, and is considered a practical and valid proxy for environmental exposure among children.

### Assessment of greenness

Greenness exposure was evaluated using the China Land Cover Dataset (CLCD), which provides annual land cover data from 1990 to 2021 at a spatial resolution of 30 × 30 m. The dataset is based on Landsat imagery processed via Google Earth Engine (GEE) and enhanced through Random Forest classification algorithms [39]. We calculated the proportion of forest and grassland within a 2-kilometer buffer around each school to estimate surrounding greenness. The average greenness coverage over the three years preceding the survey (2017–2019) was used to approximate long-term environmental exposure. This school-centered approach captures the participants’ routine environmental context and is widely applied in studies involving children.

### Assessment of physical activity

Physical activity was assessed using responses from a standardized questionnaire administered in the CNSSCH. By aligning with the Chinese Physical Activity Guidelines for Population (2021), participants were categorized into two main groups based on their activity levels: Moderate Physical Activity (< 1 h per day) and Vigorous Physical Activity (≥ 1 h per day). This classification enabled the analysis of associations between activity level and respiratory health outcomes, while also allowing for the identification of population subgroups with differing physical activity patterns.

### Assessment of VCWI

Anthropometric and lung function measurements followed standardized national protocols. Trained staff measured height and weight to the nearest 0.1 cm and 0.1 kg using calibrated equipment. FVC was assessed with an electronic spirometer that was zeroed before each session; participants stood naturally, inhaled maximally, then exhaled steadily into the mouthpiece until no further air could be expelled. Two trials were conducted per child (with no more than 15 s between attempts), and the higher FVC value (in milliliters) was recorded. Examiners corrected procedural errors in real time and cleaned spirometer tubing after every ten tests. All personnel completed standardized training and certification to ensure consistency. Body mass index (BMI) was calculated as weight (kg) divided by height squared (m²), and VCWI was calculated as FVC (ml) divided by body weight (kg). These rigorous protocols ensure reliable, high-quality data on lung function relative to body size, forming the basis for our analysis of environmental and behavioral determinants of respiratory health.

### Assessment of covariates

To control potential confounding, we included key demographic variables in the analysis. Information on age, sex, and urban/rural residence was collected through the standardized CNSSCH questionnaire. Age was treated as a continuous variable to reflect developmental variation. Sex and urbanicity were included as categorical variables to account for differences in physiological characteristics, lifestyle, and environmental conditions.

### Statistical analysis

Initially, we conducted a statistical characterization of the demographic attributes of the study participants. Continuous variables adhering to a normal distribution were expressed as the mean (± standard deviation), whereas those deviating from normality were depicted by the median (interquartile range, IQR). Categorical variables were represented by counts (percentages). We employed a linear regression model with VCWI as the dependent variable to examine its associations with PM_2.5_ and O_3_, stratified by levels of greenness (low and high) and physical activity (moderate and vigorous). This stratification aimed to elucidate trends in the relationship between VCWI and air pollutants across different subgroups.

VCWI was then dichotomized at the median value to construct a binary outcome (0 = below median, 1 = above median). We applied univariate and multivariate logistic regression models to explore associations between PM_2.5_, O_3_, greenness, and physical activity with higher VCWI, adjusting for age, sex, and urbanicity. In our analysis, all exposure variables were categorized into low and high groups using their median or mean as the cutoff, with the low group serving as the reference. Specifically, the reference groups were defined as: PM_2.5_ low group (< 41.47 µg/m³), O_3_ low group (< 146.13 µg/m³), low greenness proportion group (< 6.26%), and moderate physical activity group (< 1 h/day).

To evaluate the interactive effects among air pollutants, greenness factors, and physical activity, we adopted an additive interaction model. The interaction strength was quantified using three indices: the Relative Excess Risk due to Interaction (RERI), the Attributable Proportion (AP), and the Synergy Index (SI). In this study, greenness and physical activity were conceptualized as primary exposures of interest, in line with our central research objectives. When assessing the main effects of air pollutants (PM_2.5_ and O_3_) on VCWI, these variables were not treated as confounders and were therefore not included as covariates in the corresponding regression models. Instead, their potential roles as effect modifiers were explored through the additive interaction framework, which assessed whether greenness or physical activity modified the strength of the association between air pollution and VCWI. A positive RERI or AP, or an SI exceeding 1, indicated a synergistic interaction, where the combined effect of pollution reduction and increased greenness surpassed the sum of their individual effects. Conversely, a negative RERI or AP, or an SI below 1, suggested an antagonistic interaction, implying that pollution reduction and greenness increase may partially counteract each other. We created a new variable combining high and low exposure groups of air pollutants with high and low greenness proportions, resulting in four categories: (1) high air pollution and low greenness exposure (reference group); (2) low air pollution and low greenness exposure; (3) high air pollution and high greenness exposure; (4) low air pollution and high greenness exposure. The same approach was applied to assess the interaction between air pollution and physical activity.

For the physical activity variable, 30.5% of values were missing. We applied listwise deletion in analyses involving this variable, whereby participants with missing physical activity data were excluded only from models that included it. All other models retained the full sample. Furthermore, a sensitivity analysis was conducted to verify the consistency of our findings. This involved subgroup analysis based on participant gender and nutritional status to examine potential differences in exposure-outcome relationships. The detailed results of these subgroup analyses, including β coefficients, confidence intervals, and p-values for each factor, are presented in the Appendix (Tables S1–S7). We also tested alternative exposure timeframes by substituting the 3-year average air pollution concentrations with 1-year and 2-year averages. All analyses were conducted in R version 4.4.1, and statistical significance was defined as a two-sided p-value < 0.05.

## Results

### Demographic characteristics and environmental exposure assessment

The present study encompasses a total of 62,987 participants across eight provinces, with specific data presented in Table 1. Approximately 50% of the participants were female. The mean age was 12.04 years (SD = 3.76), with an average BMI of 19.07 kg/m² (SD = 3.77 kg/m²). Notably, 23.3% of the participants (14,662 individuals) were classified as overweight or obese. The urban-rural split was nearly equal, with 50.7% urban and 49.3% rural residents. In terms of physical function, the mean FVC was 2376.67 ml (SD = 1091.14 ml) and the mean VCWI was 52.44 ml/kg (SD = 13.96 ml/kg). Regarding physical activity intensity, 26,600 participants reported moderate activity, 17,147 reported vigorous activity, and data for 19,240 were missing. Table 2 outlines the distribution of environmental exposure factors, encompassing air pollutants (PM_2.5_ and O_3_) and greenness indicators (forest and grassland coverage), all converted into categorical variables for subsequent analysis.

**Table 1:** Demographics and characteristics of 62,987 participants

Variable	Mean ± SD or median (IQR) or *n* (%)
Age (y)	12.04 ± 3.76
Sex	
Male	31,370(49.8%)
Female	31,617(50.2%)
BMI (kg/m^2^)	19.07 ± 3.77
Nutritional status	
Normal	48,325(76.7%)
Overweight & Obesity	14,662(23.3%)
Province	
Guangdong (GDP No.1)	8212(13.0%)
Jiangsu (GDP No.2)	7848(12.5%)
Shandong (GDP No.3)	8446(13.4%)
Zhejiang (GDP No.4)	7905(12.5%)
Henan (GDP No.5)	6685(10.6%)
Sichuan (GDP No.6)	7994(12.7%)
Hubei (GDP No.7)	7847(12.5%)
Fujian (GDP No.8)	8050(12.8%)
Urbanicity	
Rural	31,057(49.3%)
Urban	31,930(50.7%)
Vital capacity	
Forced vital capacity (ml)	2376.67 ± 1091.14
Vital capacity weight index (ml/kg)	52.44 ± 13.96
Physical activity	
< 1 h/d	26,600 (42.2%)
≥ 1 h/d	17,147 (27.2%)
Missing	19,240 (30.5%)

**Table 2:** Distributions of external environmental factors

Exposure variable	Units	Spatial resolution	Median (IQR)	Mean (SD)	Range
PM_2.5_	μg/m^3^	1 km	41.467 (26.5)	42.783 (15.280)	13.733–73.000
O_3_	μg/m^3^	10 km	146.133 (39.6)	147.625 (22.758)	96.867–190.833
Forest	%	30 m * 30 m	0.483 (6.96)	6.082 (12.434)	0–70.088
Grassland	%	30 m * 30 m	0 (0.02)	0.178 (1.261)	0–19.605
Greenness	%	30 m * 30 m	0.616 (7.05)	6.259 (12.704)	0–70.138

### Associations of PM_2.5_ and O_3_ with VCWI

**Fig. 2:**
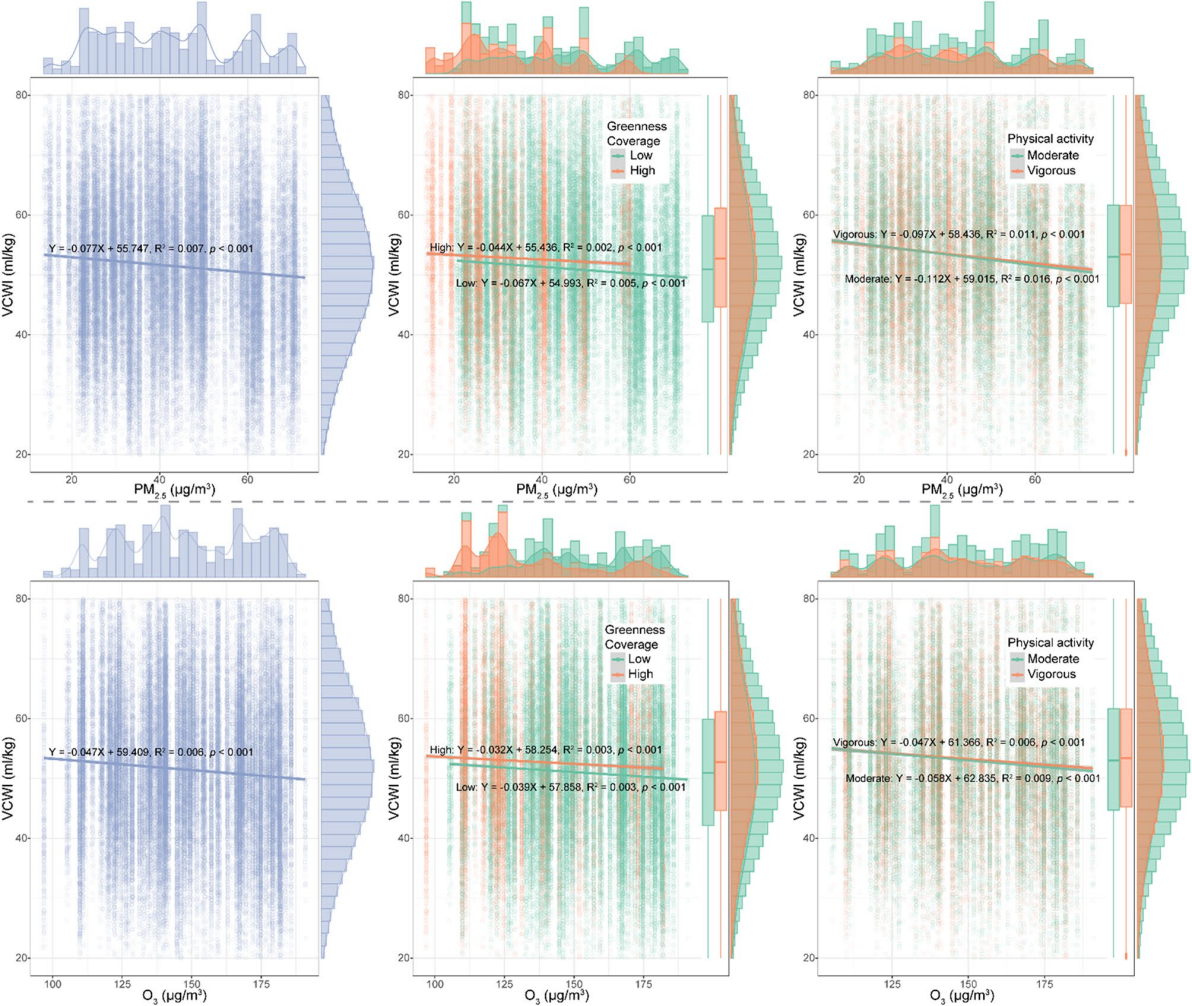
The linear regression model showing the associations of PM_2.5_ and O_3_ on VCWI

The linear regression model elucidates the relationship between VCWI and air pollutant concentrations. As illustrated in Fig. 2, an elevation in PM_2.5_ concentration is significantly associated with a decline in VCWI (regression coefficient: −0.077, *p* < 0.001), with a similar negative trend observed for O_3_ concentration (regression coefficient: −0.047, *p* < 0.001). Further stratified analysis, based on levels of greenness exposure, reveals nuanced findings. Within both high and low greenness exposure groups, VCWI decreases with increasing air pollution concentrations. However, the decline is less steep in the high greenness exposure group (PM_2.5_: − 0.044 vs. − 0.067; O_3_: − 0.032 vs. − 0.039), suggesting that higher greenness exposure may partially mitigate the adverse effects of air pollution on VCWI. Similarly, stratification by physical activity showed that participants engaging in vigorous activity experienced a weaker negative association between pollution and VCWI compared to those with moderate activity (PM_2.5_: − 0.097 vs. − 0.112; O_3_: − 0.047 vs. − 0.058). This pattern indicates that higher-intensity physical activity may also attenuate the adverse respiratory effects of air pollution.

### Effects of air pollution, greenness, and physical activity on VCWI

**Fig. 3:**
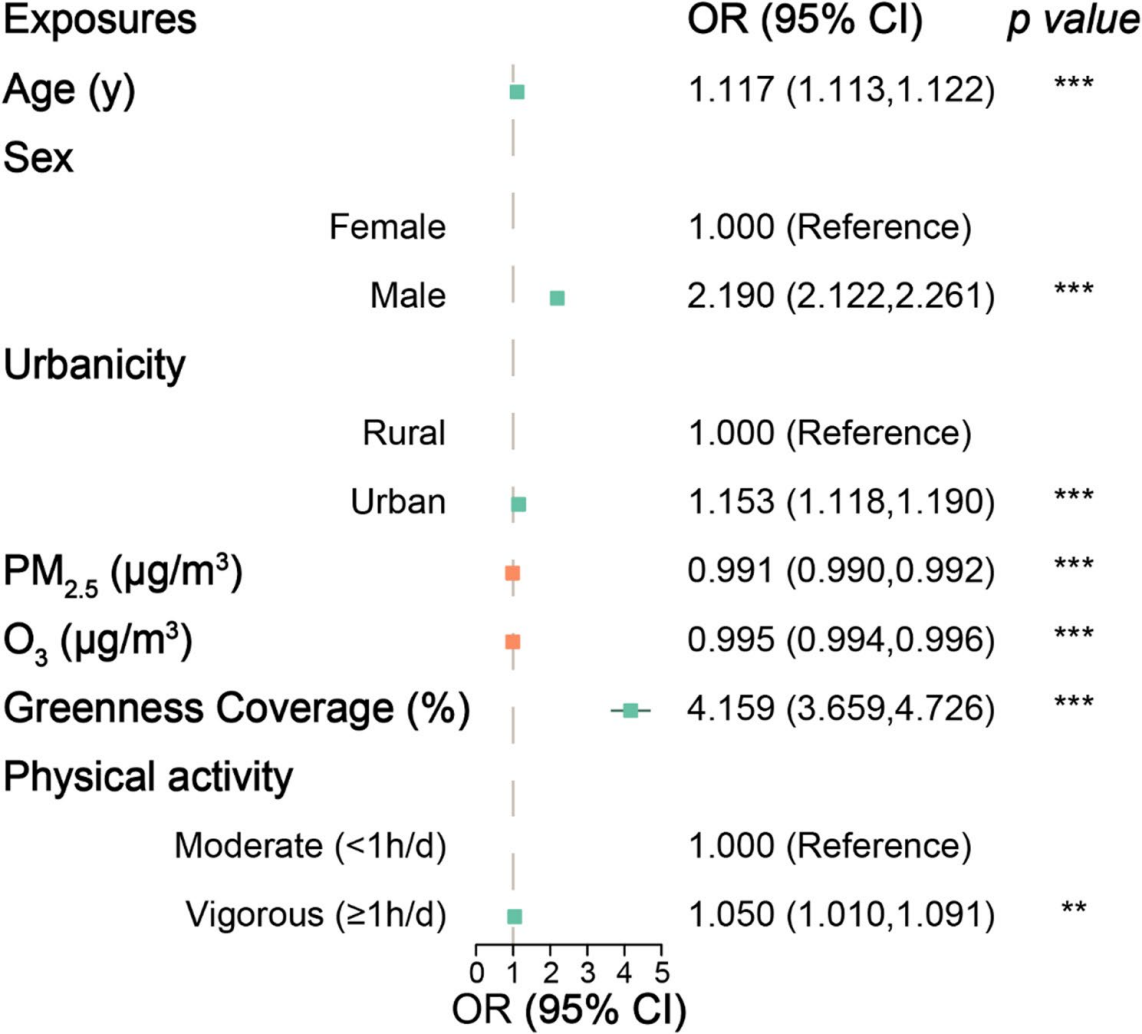
Forest plot showing effects of air pollution, greenness, and physical activity on VCWI. Note: CI, confidence interval *** *P* < 0.001, ** *P* < 0.01

Univariate logistic regression analysis revealed that age, male gender, urban residence, high greenness coverage, and vigorous physical activity were positively associated with increased VCWI. Conversely, high concentrations of PM_2.5_ and O_3_ were identified as detrimental factors, reducing VCWI (Fig. 3). In multivariate logistic regression analysis, after adjusting for covariates such as age, gender, and urbanicity (Table 3), high exposure to PM_2.5_ and O_3_ were confirmed as significant risk factors for VCWI (OR: 0.869 and 0.817, respectively). Conversely, high greenness coverage and vigorous physical activity emerged as significant protective factors (OR: 1.258 and 1.162, respectively). Furthermore, upon stratification by nutritional status, these findings persisted across all strata. Notably, the OR for vigorous activity was 1.204 in overweight/obese vs. 1.081 in normal-weight children, indicating a stronger relative benefit of exercise on VCWI among the overweight/obese subgroup.

**Table 3:** Multivariate logistic regression analysis showing the impact of different exposure variables on VCWI

Variable	Overall		Normal weight		Overweight/Obese	
	Adjusted OR (95% CI)	P value	Adjusted OR (95% CI)	P value	Adjusted OR (95% CI)	P value
PM_2.5_						
Low (<41.47μg/m^3^)	Reference		Reference		Reference	
High (≥41.47μg/m^3^)	0.869 (0.841,0.898)	***	0.942 (0.908,0.979)	**	0.921 (0.849,0.998)	**
O_3_						
Low (<146.13μg/m^3^)	Reference		Reference		Reference	
High (≥146.13μg/m^3^)	0.817 (0.790,0.844)	***	0.845 (0.814,0.878)	***	0.990 (0.912,1.074)	NS
Greenness Coverage						
Low (<6.26%)	Reference		Reference		Reference	
High (≥6.26%)	1.258 (1.213,1.304)	***	1.218 (1.168,1.271)	***	1.212 (1.106,1.328)	***
Physical activity						
Moderate (<1h/d)	Reference		Reference		Reference	
Vigorous (≥1h/d)	1.162 (1.115,1.211)	***	1.081 (1.072,1.090)	***	1.204 (1.183,1.225)	***

Adjusted for age, sex, and urbanicity

*CI* confidence interval, *NS* Not Significant

*** *P* < 0.001, ** *P* < 0.01

### Interaction between air pollutants and greenness, as well as physical activity on the improvement of VCWI

Table 4 presents an additive interaction analysis examining the combined effects of greenness, physical activity, and air pollution on VCWI improvement in children and adolescents. Using the group with low greenness and high PM_2.5_ as a reference, we found that the ORs for the other three combinations (low greenness–low PM_2.5_, high greenness–high PM_2.5_, high greenness–low PM_2.5_) were all elevated. In the interaction model, the combined effect surpassed the sum of individual effects, indicating a synergistic interaction. Specifically, the independent effects of reducing PM_2.5_ and increasing greenness on VCWI enhancement were OR 1.057 and 1.142, respectively, while their combined effect reached OR 1.321. The RERI of 0.123 (95% CI 0.019–0.221) means that there is an additional 12.3% increase in the odds of VCWI improvement attributable solely to the interaction between low PM_2.5_ and high greenness beyond their separate effects. The AP of 0.093 (95% CI 0.014–0.165) indicates that 9.3% of the combined effect can be ascribed to this synergistic interaction. The SI was 1.619 (95% CI: 0.989–2.651), which suggests a potentially synergistic effect—approximately 1.6 times greater than expected under independent action. However, as the confidence interval includes 1, this interaction did not reach statistical significance.

**Table 4:** Greenness and physical activity's interactive effects with air pollutants on VCWI

Category	N	Adjusted OR (95% CI)	P value	RERI (95% CI)	AP (95% CI)	SI (95% CI)
Greenness - PM_2.5_				0.123 (0.019, 0.221)	0.093 (0.014, 0.165)	1.619 (0.989, 2.651)
Low - High	28089	Reference				
Low - Low	17551	1.057 (1.016, 1.099)	**			
High - High	3368	1.142 (1.059, 1.231)	***			
High - Low	13979	1.321 (1.266, 1.378)	***			
Greenness - O_3_				0.378 (0.289, 0.464)	0.263 (0.204, 0.318)	7.561 (1.807, 31.647)
Low - High	27776	Reference				
Low - Low	17864	1.084 (1.042, 1.128)	***			
High - High	4459	0.973 (0.911, 1.040)	NS			
High - Low	12888	1.436 (1.374, 1.500)	***			
Physical activity - PM_2.5_				-0.047 (-0.148, 0.051)	-0.034 (-0.109, 0.036)	0.890 (0.701, 1.130)
Moderate - High	14306	Reference				
Moderate - Low	12294	1.245 (1.184, 1.309)	***			
Vigorous - High	7882	1.181 (1.115, 1.252)	***			
Vigorous - Low	9265	1.379 (1.305, 1.458)	***			
Physical activity - O_3_				-0.074 (-0.178, 0.028)	-0.052 (-0.127, 0.018)	0.853 (0.688, 1.057)
Moderate - High	14490	Reference				
Moderate - Low	12110	1.298 (1.234, 1.365)	***			
Vigorous - High	8639	1.201 (1.135, 1.271)	***			
Vigorous - Low	8508	1.425 (1.346,1.509)	***			

Adjusted for age, sex, and urbanicity

*CI* confidence interval, *NS* Not Significant

*** *P* < 0.001, ** *P* < 0.01

Similar trends were observed for greenness and O_3_, physical activity and PM_2.5_, and physical activity and O_3_: for example, the high greenness–low O_3_ combination yielded OR 1.436, and the vigorous activity–low PM_2.5_ and vigorous activity–low O_3_ combinations yielded OR 1.379 and 1.425, respectively, each showing evidence of synergy. However, no significant interaction was detected between physical activity and air pollutants, suggesting that the benefits of exercise may operate largely independently of ambient pollution levels.

## Discussion

Currently, several studies have investigated the relationship between FVC and BMI [40–42]. Similar associations have been demonstrated across diverse study populations. Steele et al. reported that obesity was associated with altered lung function independently of physical activity and fitness [43], while Berntsen et al. observed a positive association between physical activity and lung function in school-aged children [44]. However, these studies primarily focused on assessing FVC as a fundamental indicator. Most research findings suggest a positive correlation between FVC and BMI, indicating that overweight/obese children exhibit higher FVC values, which imply a greater oxygen-carrying capacity. Paradoxically, these children often demonstrate reduced physical fitness and cardiopulmonary function compared to their normal-weight counterparts [43]. Therefore, this study aims to evaluate VCWI, a more precise measure of an individual’s aerobic capacity reserve. By incorporating environmental exposure factors, such as air pollution and greenness, along with the lifestyle factor of physical activity, this study utilizes extensive data from a national survey to examine the trends in VCWI changes among children and adolescents exposed to complex environments.

Our findings reveal that concentrations of PM_2.5_ and O_3_ have potential detrimental effects on children’s respiratory reserve function. The results demonstrate a significant negative correlation between these pollutant concentrations and VCWI in children and adolescents. While research on air pollutants and lung function in pediatric populations is limited, studies in adults are more robust [45, 46]. For instance, Yang et al. [47], using data from “The China Pulmonary Health (CPH) study” across 10 provinces, confirmed a direct association between long-term increases in PM_2.5_ concentrations and declines in lung function indicators (including forced expiratory volume in 1 s (FEV1), FVC, and peak expiratory flow (PEF)), with particularly strong associations for organic matter and nitrate components in PM_2.5_. Building on this study, Niu et al. further found that long-term exposure to ozone is highly correlated with an increased risk of small airway function impairment [14]. During the warm season, for every one standard deviation increase in average ozone concentration (4.9 ppb), the FEV1/FVC ratio decreases by 0.3%, and the risk of small airway obstruction disease increases by 9%. These studies underscore that long-term exposure to air pollution is a potential risk factor for lung function impairment and the prevention and management of respiratory diseases. Additionally, a short-term exposure study conducted on children aged 9 to 12 in a Beijing school showed that exposure to high PM_2.5_ concentrations leads to a decrease in FVC, particularly in boys, with a lagged effect that is most pronounced within 0–4 days after exposure [45].

In the current study, we investigated the relationship between environmental greenness and VCWI in children, employing both univariate and multivariate logistic regression analyses. Our findings indicate that a higher proportion of environmental greenness serves as a protective factor against VCWI in both normal weight and overweight/obese children. This protective effect of greenness can be attributed to several mechanisms. First, green spaces provide accessible venues for physical activity, encouraging active lifestyles that promote respiratory development in children [28, 48]. Second, vegetation can physically remove air pollutants such as PM_2.5_ and O_3_ through processes like dry deposition and surface filtration, thereby reducing ambient concentrations and mitigating their harmful effects on lung function [49]. Third, exposure to natural environments has been shown to alleviate psychological stress, improve mental well-being, reduce noise, and help regulate microclimatic conditions (e.g., temperature and humidity), all of which may support overall physical and respiratory health [29]. In addition, this study further investigated the relationship between physical activity and VCWI among children and adolescents. The findings demonstrated that engaging in physical activity for more than one hour per day was significantly more effective in promoting an increase in VCWI compared to moderate activity of less than one hour per day. This effect was particularly evident among overweight or obese children, where vigorous physical activity exhibited a more pronounced positive impact on VCWI. One plausible explanation is that excessive adipose tissue in these individuals may restrict lung expansion and ventilation, thereby impairing pulmonary function. Regular physical activity improves FEV1 and FVC in children by strengthening respiratory muscles, optimizing lung compliance and ventilation efficiency, and reducing systemic inflammation, ultimately enhancing pulmonary function and increasing VCWI [44, 50–52]. These physiological adaptations may partially explain the observed association between vigorous physical activity and enhanced VCWI in our study [44, 53–56].

Our findings align with the recommendations in the second edition of the Physical Activity Guidelines for Americans, which advise that children and adolescents aged 6–17 engage in at least 60 min of moderate-to-vigorous physical activity each day to help reduce risk factors for chronic diseases, including obesity, insulin resistance, dyslipidemia, and elevated blood pressure [57]. The guidelines underscore the broad health benefits of regular physical activity across all age groups. Supporting evidence for the benefits of physical activity on lung function comes from studies such as Berntsen et al., which observed significant improvements in FEV1 and FVC among school-age Norwegian children who actively participated in sports and had a higher level of activity [44]. Similarly, da Silva et al. found that boys with higher levels of physical activity had above-average increases in FEV1, FVC, and PEF, speculating that physical activity might enhance lung function by increasing the amplitude and volume of thoracic movement and ventilation through regular lung expansion [58]. In the study examining the interactive effects of greening factors and environmental pollution on VCWI in children, our findings hold significant practical implications. This study reveals that in regions experiencing rapid economic growth, increasing greenness and reducing air pollution emissions can effectively protect children’s lung function, which is of great guidance for public health policy making and urban planning. Although subgroup analyses indicate that the combination of vigorous physical activity and improved air quality is associated with better VCWI outcomes, their statistical interaction was not significant.

### Limitations

This study has several limitations. First, its cross-sectional design limits causal inference and prevents the establishment of temporal relationships between exposures and outcomes. Second, although key covariates were included, potential confounders such as family socioeconomic status, indoor air pollution, dietary habits, underlying health conditions, and local microclimate factors were not available in the dataset and may influence the results. Third, the sample was restricted to provinces with GDP exceeding 4 trillion yuan, which may limit the generalizability of findings to less-developed regions. Fourth, approximately 30.5% of physical activity data were missing and addressed through listwise deletion, introducing the possibility of selection bias if missingness was not random. Fifth, physical activity was self-reported and categorized only by daily duration, lacking information on type, frequency, or intensity, which may lead to exposure misclassification. Lastly, environmental exposure was estimated using school locations due to the absence of residential addresses in the CNSSCH. While this proxy is commonly used in child health studies in China, it may not fully reflect children’s total daily exposure, especially outside school hours.

## Conclusion

In this study, utilizing comprehensive data sourced from the 2019 CNSSCH—a large, nationally representative dataset—we conducted an in-depth analysis of how environmental greenness and physical activity may buffer the adverse effects of air pollution on VCWI in children and adolescents. By integrating multiple environmental and behavioral exposures, the study offers robust and policy-relevant evidence. Notably, we found a significant inverse association between VCWI and the concentrations of PM_2.5_ and O_3_, consistent with prior research on the respiratory impacts of air pollution. Importantly, higher greenness coverage and engagement in physical activity appeared to mitigate these adverse effects, with the synergy index for greenness and PM_2.5_ exceeding unity, although without reaching statistical significance.

Our findings underscore the importance of incorporating green infrastructure and physical activity promotion into urban planning and public health strategies, especially in rapidly developing regions where both air quality challenges and opportunities for intervention are pronounced. Additionally, we observed that vigorous physical activity (≥ 1 h/day) was more strongly associated with improved VCWI than moderate activity, particularly among overweight or obese children. These results support a multifaceted approach to improving respiratory health in youth populations.

## Supplementary Information

Below is the link to the electronic supplementary material.

Supplementary Material 1

## Data Availability

The datasets generated and/or analyzed during the current study are not publicly available due to institutional data sharing policies, but are available from the corresponding author on reasonable request.
