# Supplementary material for: Environmental greenness, physical activity, and their synergistic effects on vital capacity weight index in children and adolescents exposed to PM2.5 and O3 in economically developed provinces of China
Source: BMC Public Health. 2025 Oct 9;25:3427. doi: 10.1186/s12889-025-24439-9 (PMC12512696; doi:10.1186/s12889-025-24439-9)
Supplement: Supplementary file 1 — Supplementary Material 1 [file 12889_2025_24439_MOESM1_ESM.docx]

**Appendix**

**Title:** Environmental Greenness, Physical Activity, and Their Synergistic Effects on Vital Capacity Weight Index in Children and Adolescents Exposed to PM2.5 and O3 in Economically Developed Provinces of China

**Authors:** Zhiying Song, Xinli Song, Li Chen, Jianuo Jiang, Yi Zhang, Jieyu Liu, Ruolin Wang, Yang Qin, Ziqi Dong, Tongjun Guo, Wen Yuan, Peijin Hu, Tianjiao Chen, Guangrong Zhu, Jun Ma, Yanhui Dong**^‡^**, Yi Song**^‡^**

**Affiliations:**

Institute of Child and Adolescent Health, School of Public Health, Peking University/National Health Commission Key Laboratory of Reproductive Health, Beijing, China;

**‡, Correspondence:**

**Yanhui Dong** and **Yi Song**, Institute of Child and Adolescent Health & School of Public Health, Peking University, No. 38 Xueyuan Rd, Haidian District, Beijing 100191, China, Email: [dongyanhui@bjmu.edu.cn](mailto:dongyanhui@bjmu.edu.cn); [songyi@bjmu.edu.cn](mailto:songyi@bjmu.edu.cn).

**Supplemental Information**

**Table S1.** Association between physical activity(4-category) and VCWI

**Table S2.** Association between PM_2.5_ (3-category) and VCWI

**Table S3.** Association between O_3_ (3-category) and VCWI

**Table S4.** Association between Forest Coverage (3-category) and VCWI

**Table S5.** Association between Grass Coverage (3-category) and VCWI

**Table S6.** Association between Green Land Coverage (3-category) and VCWI

**Table S7.** Association of environmental factors and physical exercise with VCWI by nutritional status (Normal weight vs. Overweight & Obesity)

**Table S1.** Association between physical activity(4-category) and VCWI

| Physical exercise | β (95%CI) | p value |
| --- | --- | --- |
| ≤0.5h/d | Reference | |
| 0.5~1h/d | -0.043 (-0.417, 0.330) | 0.820 |
| 1~2h/d | 0.883 (0.477, 1.288) | <0.001 |
| ≥2h/d | 1.261 (0.735, 1.788) | <0.001 |

Note: The model was adjusted for demographic information covariates, including age, sex, and urbanicity.

CI, confidence interval.

**Table S2.** Association between PM_2.5_ (3-category) and VCWI

| PM_2.5_ (μg/m³) | β (95%CI) | p value |
| --- | --- | --- |
| 1st (≤33.4) | Reference | |
| 2nd (33.4~49.5) | 0.244 (–0.007, 0.496) | 0.057 |
| 3rd (≥49.5) | –2.472 (–2.725, –2.220) | <0.001 |
| Continuous | –0.081 (–0.088, –0.075) | <0.001 |

Note: Cut-points at the 33.3rd (33.4μg/m³) and 66.7th (49.5μg/m³) percentiles.

The model was adjusted for demographic information covariates, including age, sex, and urbanicity.

CI, confidence interval.

**Table S3.** Association between O_3_ (3-category) and VCWI

| O_3_ (μg/m³) | β (95%CI) | p value |
| --- | --- | --- |
| 1st (≤136.8) | Reference | |
| 2nd (136.8~160.1) | 0.084 (–0.168, 0.336) | 0.513 |
| 3rd (≥160.1) | –2.727 (–2.979, –2.476) | <0.001 |
| Continuous | –0.051 (–0.056, –0.047) | <0.001 |

Note: Cut-points at the 33.3rd (136.8μg/m³) and 66.7th (160.1μg/m³) percentiles.

The model was adjusted for demographic information covariates, including age, sex, and urbanicity.

CI, confidence interval.

**Table S4.** Association between Forest Coverage (3-category) and VCWI

| Forest coverage | β (95%CI) | p value |
| --- | --- | --- |
| Low (≤25%) | Reference | |
| Medium (25%~50%) | 3.431 (2.981, 3.880) | <0.001 |
| High (≥50%) | 6.655 (5.990, 7.320) | <0.001 |
| Continuous | 11.098 (10.270, 11.925) | <0.001 |

Note: Defined a priori as Low≤25%, Medium 25%~50%, High≥50%.

The model was adjusted for demographic information covariates, including age, sex, and urbanicity.

CI, confidence interval.

**Table S5.** Association between Grass Coverage (3-category) and VCWI

| Forest coverage | β (95%CI) | p value |
| --- | --- | --- |
| Low (≤1%) | Reference | |
| Medium (1%~5%) | 1.243 (0.214, 2.272) | 0.018 |
| High (≥5%) | 3.705 (1.744, 5.665) | <0.001 |
| Continuous | 21.015 (12.738, 29.292) | <0.001 |

Note: Defined a priori as Low≤1%, Medium 1%~5%, High≥5%.

The model was adjusted for demographic information covariates, including age, sex, and urbanicity.

CI, confidence interval.

**Table S6.** Association between Green Land Coverage (3-category) and VCWI

| Forest coverage | β (95%CI) | p value |
| --- | --- | --- |
| Low (≤25%) | Reference | |
| Medium (25%~50%) | 3.410 (2.950, 3.869) | <0.001 |
| High (≥50%) | 6.377 (5.748, 7.007) | <0.001 |
| Continuous | 21.015 (12.738, 29.292) | <0.001 |

Note: Defined a priori as Low≤25%, Medium 25%~50%, High≥50%.

The model was adjusted for demographic information covariates, including age, sex, and urbanicity.

CI, confidence interval.

**Table S7.** Association of environmental factors and physical exercise with VCWI by nutritional status (Normal weight vs. Overweight & Obesity)

| Variable | Normal weight  (β, 95% CI) | p value | Overweight & Obesity  (β, 95% CI) | p value |
| --- | --- | --- | --- | --- |
| PM_2.5_ | -0.058 (-0.066, -0.051) | <0.001 | -0.043 (-0.054, -0.031) | <0.001 |
| O_3_ | -0.046 (-0.051, -0.041) | <0.001 | -0.015 (-0.023, -0.008) | <0.05 |
| Forest coverage | 10.065 (9.175, 10.956) | <0.001 | 7.666 (6.093, 9.239) | <0.001 |
| Grass coverage | 27.984 (18.787, 37.181) | <0.001 | 18.754 (4.858, 32.650) | <0.05 |
| Green land coverage | 9.931 (9.058, 10.804) | <0.001 | 7.468 (5.939, 8.996) | <0.001 |
| Physical exercise | 0.355 (0.192, 0.518) | <0.001 | 0.811 (0.544, 1.078) | <0.001 |

Note: The model was adjusted for demographic information covariates, including age, sex, and urbanicity.

CI, confidence interval.
